# Random Mutagenesis by Insertion of Error-Prone PCR Products to the Chromosome of *Bacillus subtilis*

**DOI:** 10.3389/fmicb.2020.570280

**Published:** 2020-11-13

**Authors:** Bin Ye, Yu Li, Qing Tao, Xiaoliang Yao, Minggen Cheng, Xin Yan

**Affiliations:** Key Laboratory of Agricultural Environmental Microbiology, Ministry of Agriculture, College of Life Sciences, Nanjing Agricultural University, Nanjing, China

**Keywords:** epPCR, chromosomal integration, large library, directed evolution, *Bacillus subtilis*, optimization of protein expression

## Abstract

*Bacillus subtilis* is an attractive host for the directed evolution of the enzymes whose substrates cannot be transported across cell membrane. However, the generation of a mutant library in *B. subtilis* suffers problems of small library size, plasmid instability, and heterozygosity. Here, a large library of random mutant was created by inserting error-prone PCR (epPCR) products to the chromosome of *B. subtilis*. Specifically, the epPCR product was fused with flanking regions and antibiotic resistant marker using a PCR-based multimerization method, generating insertion construct. The epPCR product was integrated into the chromosome via homologous recombination after the insertion construct was transformed into the supercompetent cells of *B. subtilis* strain SCK6. The transformation efficiency of the insertion construct was improved through co-expressing homologous recombination-promoting protein NgAgo, raising the number of competent cells, and increasing the length of flanking regions. A library containing 5.31 × 10^5^ random mutants was constructed using per μg insertion construct, which is sufficient for directed evolution. The library generation process was accomplished within 1 day. The effectiveness of this method was confirmed by improving the activity of Methyl Parathion Hydrolase (MPH) toward chlorpyrifos and by enhancing the secretion level of MPH in *B. subtilis*. Taken together, the present work provides a fast and efficient method to integrate epPCR products into the chromosome of *B. subtilis*, facilitating directed evolution and expression optimization of target proteins.

## Introduction

Directed evolution has been proved to be a powerful tool to improve the activity, stability, and substrate specificity of enzymes (Wong et al., 2004, 2007; Roodveldt et al., 2005; Romero and Arnold, 2009; Goldsmith and Tawfik, 2017; Zeymer and Hilvert, 2018). This tool involves two crucial steps: the generation of a library containing sufficient gene variants, and high-throughput screening of library members with desired properties (Wong et al., 2006; Packer and Liu, 2015). Because of its high transformation efficiencies, rapid growth rates, well-established manipulation approaches (Crameri et al., 1996), *Escherichia coli* is generally employed as the host organism for library creation. However, proteins are usually expressed in cytoplasm in *E. coli*, making the screening of the library more difficult if the substrate of the protein cannot be transported into the cell. Therefore, the organisms such as *Bacillus subtilis* and *Pichia pastoris* that can secret proteins into medium have been developed as alternative hosts for library generation (Reetz and Carballeira, 2007; Wang et al., 2012; Li Y. X. et al., 2017; Liu et al., 2017; Jiang et al., 2019).

*B. subtilis* is an important industrial host for the production of various recombinant proteins due to its GRAS (generally recognized as safe) status, excellent protein secretion ability and mature fermentation processes (Marcus et al., 2004; Terpe, 2006; Schumann, 2007; Yang et al., 2011; Commichau et al., 2014). Although *B. subtilis* has well-developed genetic manipulation tools, its transformation frequency is still much lower than that of *E. coli*. Thus, the library of random mutants generated through digestion and ligation is too small to fulfill the needs of directed evolution in *B. subtilis*. Several strategies have been adopted to address this limitation. A routine strategy is first constructing the library of variants in *E. coli* and then transferring the library members into *B. subtilis*, which is time-consuming and of low efficiency (You and Arnold, 1996; Zhao and Arnold, 1999; Caspers et al., 2010). Melnikov and Youngman (1999) attempted to clone the epPCR product via marker-replacement recombination with a structurally similar helper plasmid resident in the transformation recipient. But they found that it was difficult to recover > 10^3^ transformants/μg of epPCR product. Given the phenomenon that multimeric plasmid has much higher (approximately three orders of magnitude) transformation frequency than that of the monomeric plasmid in *B. subtilis* (Canosi et al., 1978), Shafikhani et al. (1997) generated large libraries of random mutants (3 × 10^6^) in *B. subtilis* by PCR-based plasmid multimerization method. In this method, epPCR product was fused with a linearized vector through PCR extension to generate linear plasmid multimer which could be converted to circular form through homologous recombination after entering the cell. However, each cell may take several variants, decreasing screening efficiency. Increasing the number of competent cells is an effective method to create a larger library. To this end, Zhang and Zhang (2011) constructed a *B. subtilis* strain SCK6 whose competence can be induced by controlling the expression of master regulator ComK artificially. The procedure for preparing the supercompetent cell of strain SCK6 was further improved by Li X. Z. et al. (2017). The transformation frequencies of multimeric plasmid, monomeric plasmid, and integration plasmid into strain SCK6 could reach 10^7^, 10^4^, and 10^5^ transformants/μg DNA, respectively.

This study aims to construct a large mutant library by inserting epPCR product into the chromosome of *B. subtilis*, which will solve the problems of plasmid instability and heterozygosity faced by multi-copy plasmid mediated strategies (Melnikov and Youngman, 1999; Zhang and Zhang, 2011; You et al., 2012). The library construction procedure was accomplished within 1 day and the generated library had sufficient mutants (> 10^5^) for direct evolution.

## Materials and Equipment

### Bacterial Strains, Primers, and Growth Conditions

The *B. subtilis* SCK6 strain was provided by Dr. Daniel R. Zeigler from the *Bacillus* Genetic Stock Center (BGSC). The oligonucleotide synthesis and DNA sequencing were performed by Sangon Biotech Co., Ltd. (Shanghai, China). *B. subtilis* strains were cultivated in Lysogeny Broth (LB) (Bertani, 2004) medium, YN medium (Li X. Z. et al., 2017) or 2 × Super-Rich (2 × SR) (Song et al., 2017) medium. The LB medium consists of 1% tryptone, 0.5% yeast extract, and 0.5% NaCl. The YN medium is composed of 0.7% yeast extract and 1.8% nutrient broth. The 2 × SR medium (3% tryptone, 5% yeast extract, and 0.6% K_2_HPO_4_, pH 7.2) was used for fermentation. The solid medium was obtained by adding 15 g/L agar to the liquid medium. Unless otherwise indicated, the final concentrations of antibiotics were as follows (mg/L): zeocin (Zeo), 20; erythromycin (Em), 5; chloromycetin (Cm), 5. The inoculums (1%, V/V) were transferred into 250 mL flasks containing 30 mL 2 × SR medium and incubated at 37°C with shaking at 200 rpm. Chlorpyrifos (> 99%) was purchased from the Macklin Biochemical Technology Co., Ltd. (Shanghai, China).

### Construction of *B. subtilis* SCK6A

*B. subtilis* SCK6A was constructed by inserting the *xylose*-inducible promoter *P*_*xylA*_ controlled *NgAgo*-D663A-D738A (Fu et al., 2019) at the *thrC* locus in the chromosome of *B. subtilis* SCK6. The left flanking region (LF), *xylR*-*P_*xylA*_*, and right flanking region (RF) were amplified from *B. subtilis* SCK6 genomic DNA using the primer pairs P11/P12, P15/P16, and P19/P20, respectively. The Cm^*R*^ gene was amplified with the primer pair P13/P14 using plasmid pNW33N (BGSC) as the template. The *NgAgo*-D663A-D738A gene was amplified with the primer pair P17/P18 using plasmid pHT-XCR6 (MolecularCloud plasmid sharing platform Cat.no: MC_0068418) as the template. These five fragments were fused by overlap PCR in the following order: LF, Cm^*R*^, *xylR*-*P*_*xylA*_, *NgAgo*, and RF. The PCR-product was directly transformed into strain SCK6, and the transformants were selected on LB plates containing Cm.

### Construction of Methyl Parathion Hydrolase (MPH) Secretion Strain

Four DNA fragments including *P_*cry*3A_* promoter (Adams et al., 1994), the coding region of the signal peptide (SP_AprE_), the MPH-encoding gene *mpd* (Zhang et al., 2005), and the T1T2 transcription terminator (Hartl et al., 2001) were fused by overlap PCR (Shevchuk et al., 2004) using the primers listed in Table 1, generating the MPH expression cassette *P_*cry*3A_*-*mpd*. The expression cassette was then fused with flanking regions of *amyE* and Zeo resistant marker (Zeo^R^), producing the insertion construct, which was then transformed into the competent cells of strain SCK6 and selected by Zeo. *P_*cry*3A_*-*mpd* was finally integrated at the locus of *amyE* in the chromosome of strain SCK6, generating strain BPC1.

**TABLE 1:** Primers used in this study.

Primers	Sequence (5′–3′)	Purpose
P1	tgaactttatctgagaatagtcaatcttcggaaatcccaggtggc	For the construction of multimer of insertion construct (LF-Ab^*R*^-GOI-RF)
P2	catttttcttcctccctttcttatcataatacataattttcaaactg	
P3	cagtttgaaaattatgtattatgataagaaagggaggaagaaaaatg	
P4	gccacctgggatttccgaagattgactattctcagataaagttca	
P5	cagcgcaaatgctcccgctatcatcgagctccagcatccttgcagtcttcatatg	
P6	catatgaagactgcaaggatgctggagctcgatgatagcgggagcatttgcgctg	
P7	tttggaaagcgaggga	For the construction of variant T47C
P8	ctgaacgccatcgtaaagattgacgttaacgcaaacaacaaacttatc	
P9	gataagtttgttgtttgcgttaacgtcaatctttacgatggcgttcag	
P10	cgttggttgtatccgtgt	
P11	agccgacactgcttcctg	For the construction of strain SCK6A
P12	catcatctgtatgaatcaaatcgcggccttcaatgcggtaagggttg	
P13	caacccttaccgcattgaaggccgcgatttgattcatacagatgatg	
P14	cggcaaccgagcgttctgaaactcacattaattgcgttgcg	
P15	cgcaacgcaattaatgtgagtttcagaacgctcggttgccg	
P16	catggatcccacctcctttaattgggactagtttggaccatttgtc	
P17	gacaaatggtccaaactagtcccaattaaaggaggtgggatccatg	
P18	caaagccgcgcattttcggaaggccttagaggaatccgacattagactcgaac	
P19	gttcgagtctaatgtcggattcctctaaggccttccgaaaatgcgcggctttg	
P20	agaatcgttgggcctgct	
P21	cgcacctgcggtgctgcagcctgagcagacatgttgctgaacgcc	For the construction of variant G81T
P22	ggcgttcagcaacatgtctgctcaggctgcagcaccgcaggtgcg	
P23	gcggcggacttgccgtcgatgtcgagctgggtcgtgacgctggggtcgtc	For the construction of variant T806A
P24	gacgaccccagcgtcacgacccagctcgacatcgacggcaagtccgccgc	
P25	gccttcttgcgctccaccgcgacggacttgccgtcgatgtcgagc	For the construction of variant C821T
P26	gctcgacatcgacggcaagtccgtcgcggtggagcgcaagaaggc	
P27	gatgtggccgatgccggggaacgacaggtggctcgccgcgatcag	For the construction of variant C892T
P28	ctgatcgcggcgagccacctgtcgttccccggcatcggccacatc	
P29	gagtagttcaccggcacgaaatggtagcccttgccttcggcgc	For the construction of variant G938A
P30	gcgccgaaggcaagggctaccatttcgtgccggtgaactactc	

### Transformation of *B. subtilis*

The *B. subtilis* SCK6 and SCK6A strains were inoculated into 4 mL of YN medium with 5 mg/L erythromycin in a test tube. The cells were cultivated at 37°C with shaking at 220 rpm overnight (∼12 h). The culture was diluted to an optical density (*OD*_600 nm_ = 1.0) in a fresh YN medium containing 1.5% (w/v) xylose and then was cultivated for 2 h. The resulting cell cultures were the supercompetent cells that were transformed. 100 ng of DNA was mixed with different volumes of the supercompetent cells in a 1.5 mL eppendorf tube and cultivated at 37°C with shaking at 220 rpm for 90 min.

### Screening of the Mutant Library

The transformants were selected on LB plates containing Zeo and the colonies were then transferred to LB plates containing 50 mg/L chlorpyrifos using a sterile toothpick. Strain BPC1was used as the control. After incubation at 37°C for 12 h, the MPH activity of each colony was determined according to the size of transparent halos. The mutants with larger transparent halos were selected for further verification.

### MPH Purification and Chlorpyrifos-Hydrolyzing Activity Assay

The MPH purification was performed following the method described by Zhao et al. (2019). Briefly, the cells harboring MPH expression cassette were cultivated in a 2 × SR medium for 36 h at 37°C. The supernatant of the cultures was used for purification of the C-terminal His-tagged MPH through Ni-NTA affinity chromatography according to the manufacturer’s instructions (Sangon Biotech Co., Ltd., Shanghai, China). Protein content was measured by the BCA Kit (Shanghai, Sangon Biotech Co., Ltd., China). The target proteins were analyzed by SDS-PAGE and the gel was stained with Coomassie brilliant blue R250. The activity of MPH toward chlorpyrifos was measured as described previously (Xie et al., 2014).

### Site-Directed Mutagenesis

Each mutation was introduced into wildtype *P_*cry*3A_*-*mpd* expression cassette through overlap PCR (Shevchuk et al., 2004). The genomic DNA of strain BPC1 was used as the template and the primers were listed in Table 1. The PCR product was directly transformed into strain SCK6, generating the mutants.

## Methods

**FIGURE 1:**
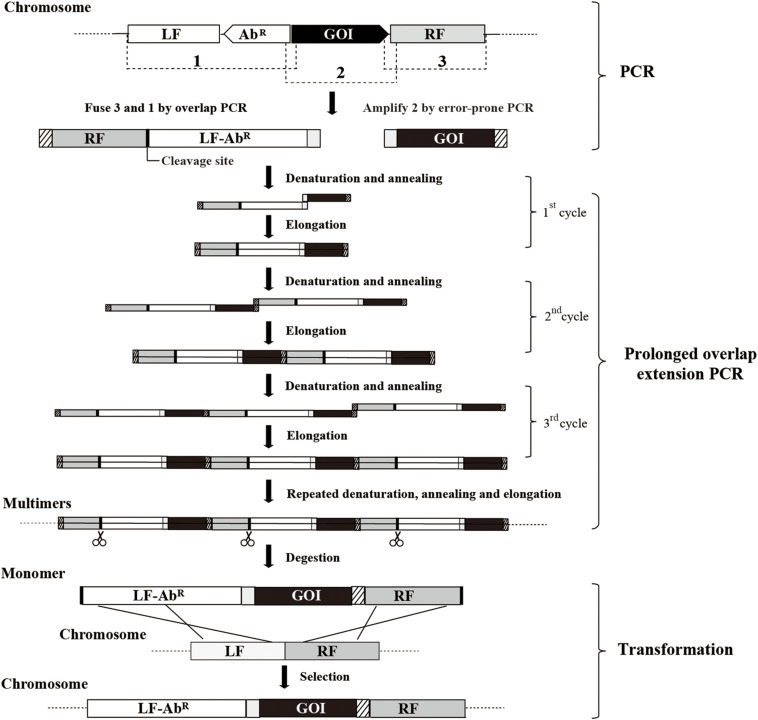
The scheme for insertion of epPCR products to the chromosome of *B. subtilis.* Firstly, fragment RF-LF-Ab^*R*^ is generated by overlap PCR and fragment GOI is amplified by error-prone PCR (epPCR). Secondly, the DNA multimer is formed by prolonged overlap extension PCR using fragments RF-LF-Ab^*R*^ and GOI. Thirdly, the DNA multimers are digested to monomer (LF-Ab^*R*^-GOI-RF) at the cleavage site introduced between RF and LF. Finally, the insertion construct is transformed into competent cells. LF, left flanking region; Ab^*R*^, antibiotic resistant marker; GOI, gene of interest; RF, right flanking region; scissors, restriction endonuclease digestion.

A scheme summarizing the methodology for random mutagenesis by insertion of PCR products to the chromosome is illustrated in Figure 1. The epPCR product is firstly assembled into an insertion construct consisting of the left flanking region (LF), antibiotic resistant marker (Ab^*R*^), epPCR product, and right flanking region (RF); then, after the insertion construct is transformed into *B. subtilis* competent cells, the epPCR product is inserted into the chromosome of *B. subtilis* through homologous recombination. The efficient assembly of the insertion construct is crucial in this method. The plasmid multimerization method (Shafikhani et al., 1997; You et al., 2012) is employed to assemble the insertion construct. First, LF, RF, and Ab^*R*^ are fused through overlap PCR (Shevchuk et al., 2004) generating fragment RF-LF-Ab^*R*^; notably, RF is put ahead of LF and a cleavage site of restriction enzyme is introduced between RF and LF. The gene of interest (GOI) is amplified by epPCR. The left end of GOI overlapped (40–50 bp) with the right end of RF-LF-Ab^*R*^, while the right end of the GOI overlapped (40–50 bp) with the left end of RF-LF-Ab^*R*^. Then, the equal molar amount of GOI and RF-LF-Ab^*R*^ are mixed, and the multimer of insertion construct (LF-Ab^*R*^-GOI-RF) is generated by prolonged overlap extension PCR. Finally, the multimer is cut at the cleavage site between RF and LF, releasing the monomer of the insertion construct.

After the MPH mutant library was generated, following the above scheme, the RF of the *amyE* locus was amplified from the genomic DNA of strain BPC1 using primer pair P1/P5. Another fragment containing LF, Zeo^*R*^ and *P_*cry*3A_* was amplified as well using primer pair P6/P2. These two fragments were fused by overlap PCR (Shevchuk et al., 2004) using primer pair P1/P2, resulting in fragment RF-LF-Zeo^*R*^-*P_*cry*3A_*. The cleavage site *Sac*I was introduced between RF and LF. The coding region of SP_*AprE*_ and MPH was amplified by epPCR with primer pair P3/P4 using the Starmut Random Mutagenesis Kit (GenStar, Beijing, China). The PCR reaction solution with a total volume of 50 μL contained 0.02 ng/μL template, 25 μL 2× buffer, 3 μL StarMut Enhancer, 0.4 mM primer pair P3/P4. The PCR was conducted as follows: initial denaturation at 95°C for 2 min and subsequent steps of denaturation at 94°C for 30 s, annealing at 56°C for 1 min, and extension at 72°C for 1 min for 30 cycles in total. The overlap of 47 bp between the left end of the epPCR product and the right end of RF-LF-Zeo^*R*^-*P_*cry*3A_* was introduced through primers P4 and P1, and the overlap of 45 bp between the right end of the epPCR product and the left end of RF-LF-Zeo^*R*^-*P_*cry*3A_* was introduced through primers P3 and P2. To generate the multimer of fragment LF-Zeo^*R*^-*P_*cry*3A_*-*mpd*-RF, each 50 μL reaction system contained 0.2 mM dNTP, an equal molar amount of RF-LF-Zeo^*R*^-*P_*cry*3A_* and epPCR product (∼30 pmol for each fragment), and 0.04 U/μL Phusion polymerase without the addition of primers. The prolonged overlap extension PCR was conducted as follows: denaturation at 98°C for 30 s; 30 cycles of denaturation at 98°C for 15 s, annealing at 60°C for 15 s, and extension at 72°C at a rate of 2 kb/min; followed by 72°C extension for 10 min. The multimer product was digested with *Sac*I to release the monomer of fragment LF-Zeo^*R*^-*P_*cry*3A_*-*mpd*-RF which was then transformed into the competent cells of strain SCK6.

## Results

### Development of the Random Mutagenesis System

**FIGURE 2:**
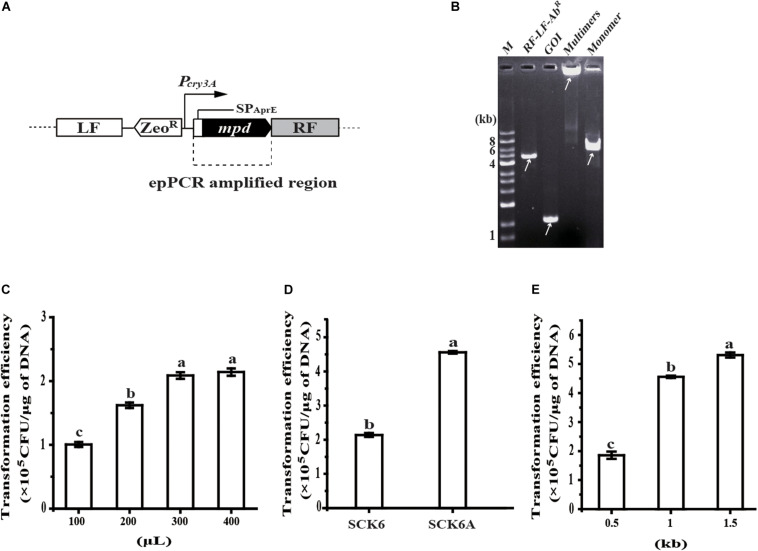
Development of the random mutagenesis system. **(A)** Construction of MPH secretion strain BPC1. Promoter *P_cry3A_* controlled transcription of *mpd* and the signal-peptide of AprE (SP_*AprE*_) mediated the secretion of the Methyl Parathion Hydrolase (MPH). **(B)** Analysis of the insertion construct assembly process by agarose gel electrophoresis. The arrow indicates the position of the target DNA. **(C)** The effect of the competent *B. subtilis* SCK6 cell amount on transformation efficiency of the insertion construct. The amount of the insertion construct was 100 ng and the flanking region at each side was 1 kb. **(D)** The transformation efficiency of the insertion construct in strains SCK6 and SCK6A. The volume of competent cells was 400 μL, the amount of the insertion construct was 100 ng and each flanking region was 1 kb. **(E)** The effect of the flanking region size on the transformation efficiency of the insertion construct. The amount of the insertion construct was 100 ng and the volume of competent *B. subtilis* SCK6A cells is 400 μL. All data were collected from at least three biological replicates and are shown as the mean ± SD. Bars indicated by the same letter are not significantly different (*P* > 0.05, evaluated by Duncan’s test).

The random mutagenesis method was tested by the directed evolution of Methyl Parathion Hydrolase (MPH) to improve its activity toward chlorpyrifos, a pesticide contaminant that is often detected in food and the environment (Lu et al., 2013). To begin with, the secretion expression of MPH was realized by inserting MPH expression cassette at the *amyE* locus in the chromosome of *B. subtilis*; the transcription of *mpd* is driven by promoter *P_*cry*3A_*, and the secretion of MPH is mediated by signal-peptide of AprE (SP_*AprE*_) (Figure 2A). Then, the coding region of SP_*AprE*_ and MPH is amplified by epPCR, and another fragment of RF-cleavage site (*Sac*I)-LF-Zeo^*R*^-*P_*cry*3A_* is generated via overlap PCR. After multimerization, the product was converted to a monomer of the insertion construct (LF-Zeo^*R*^-*P_*cry*3A_*-*mpd*-RF) by *Sac*I digestion, which was confirmed by agarose gel electrophoresis. As shown in Figure 2B, neither of the two fragments (RF-LF-Zeo^*R*^-*P_*cry*3A_* and epPCR product) could be detected on the gel after multimerization, indicating most of the two fragments were transformed into multimers. The *Sac*I digestion product matched the size of insertion construct, which was further verified by DNA sequencing.

To enhance the transformation efficiency of the insertion construct, the amount of competent cells was first raised for each transformation reaction. Typically, when 400 μL competent cells were mixed with 100 ng DNA, the transformation efficiency reached 2.14 × 10^5^ CFU/μg DNA, 2.12-fold than that of 100 μL competent cells (Figure 2C). As protein NgAgo could enhance homologous recombination in bacteria (Fu et al., 2019; Wu et al., 2020), the gene encoding a mutant of *NgAgo* was then inserted into the chromosome of strain SCK6, under the control of xylose-inducible promoter *P*_*xylA*_. The transformation efficiency of the insertion construct in the NgAgo-expressing host (strain SCK6A) was about 2.13-fold that in strain SCK6 (Figure 2D). We found that the transformation efficiency was positively correlated with the length of the flanking region. The flanking region of 0.5, 1, and 1.5 kb on each side led to the transformation efficiency of 1.85 × 10^5^, 4.56 × 10^5^, and 5.31 × 10^5^ CFU/μg DNA (Figure 2E), respectively.

### Screening of the Mutant Library of MPH Variants

**FIGURE 3:**
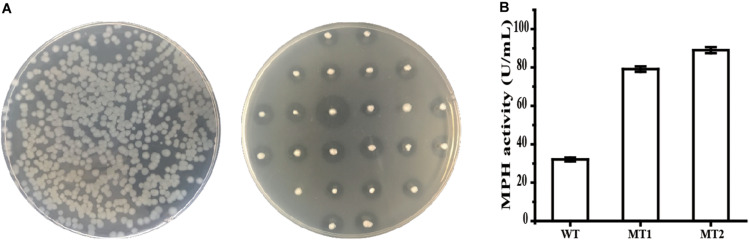
Screening of the library of MPH variants. **(A)** Screening of the library on an LB plate containing 50 mg/L chlorpyrifos. Frist, transformants were selected on LB plates containing Zeocin. Then, the colonies were transferred to LB plates containing 50 mg/L chlorpyrifos using a sterile toothpick. When chlorpyrifos are hydrolyzed by MPH, a transparent halo forms around the colony. **(B)** The extracellular activities of MPH in the supernatant of strain BPC1 and its mutants. All data were collected from at least three biological replicates and are shown as the mean ± SD.

The flanking region of 1-kb at each side of the insertion construct was used to generate a library of *mpd* variants using strain SCK6A. Ten clones were randomly selected, and the DNA sequencing result of the SP_*AprE*_ and MPH coding region shows that each mutant harbored 1–5 mutations, with an overall mutation rate of 0.31%. As shown in Figure 3A, the mutants were grown on an LB plate containing 50 mg/L chlorpyrifos. A transparent halo formed around the colony when chlorpyrifos was hydrolyzed by MPH (Xie et al., 2014). An enlarged halo indicated enhanced MPH activity, which may be caused by improved catalytic efficiency or increased MPH amount. Two mutants, named MT1 and MT2, which formed significantly larger transparent halos, were screened from about 12,000 colonies. When grown in a liquid medium, the growth curves of both mutants were similar to that of the parent strain BPC1 (data not shown), but the maximum extracellular MPH activity of mutants MT1 and MT2 were 2.47- and 2.77-fold that of strain BPC1 (Figure 3B), respectively.

### Identification of the Effect of Each Mutation on MPH

**FIGURE 4:**
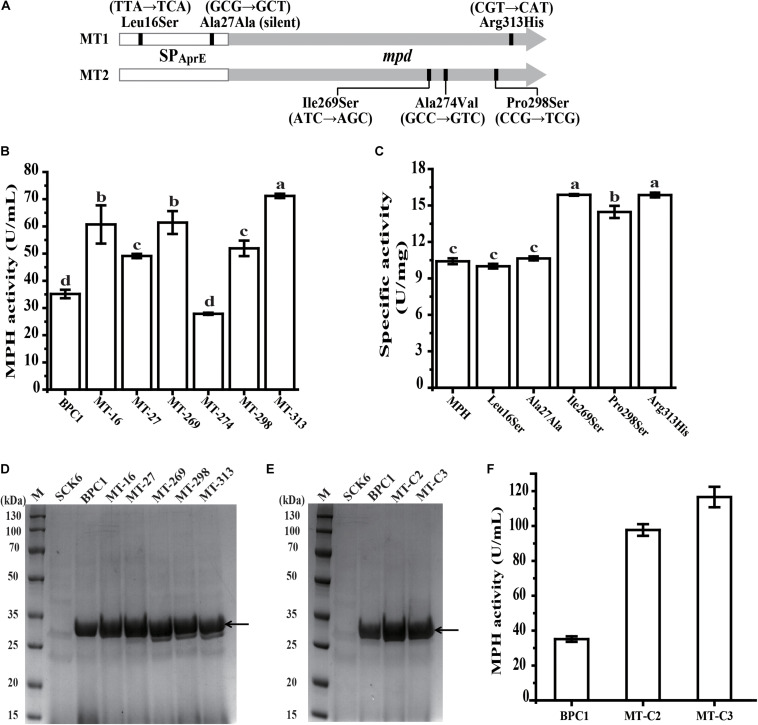
Identification of the effect of each mutation on MPH. **(A)** Base mutations in *mpd* variants. The vertical line indicates the base mutations in the variants. **(B)** The extracellular activities of MPH in the supernatant of strain BPC1 and six mutants with a single mutation. **(C)** Specific activity of wild-type MPH and the five variants with a single mutation. **(D)** SDS-PAGE analysis of extracellular MPH in the supernatant of the strain BPC1 and its mutants. **(E)** SDS-PAGE analysis of extracellular MPH in the supernatant of the mutants MT-C2 and MT-C3. Mutant MT-C2 harbors mutations T47C and T806A, and mutant MT-C3 contains mutations of T47C, T806A, and G938A. The strains were grown on 2 × SR medium and incubated at 37°C with shaking at 200 rpm for 36 h. Equal amounts (20 μL) of culture supernatant were loaded into each lane. **(F)** The extracellular activities of MPH in the supernatant of strain BPC1, MT-C2, and MT-C3. M, protein markers. The arrow indicates the position of the target band. All data were collected from at least three biological replicates and are shown as the mean ± SD. Bars indicated by the same letter are not significantly different (*P* > 0.05, evaluated by Duncan’s test).

The SP_*AprE*_ and MPH coding region in mutant MT1 and MT2 were sequenced individually. The mutation sites are shown in Figure 4A. The mutant MT1 contained three mutations, with two (T47C and G81T) located in the SP_*AprE*_ coding region and one (G938A) located in MPH coding region. Among these three mutations, two led to amino acid change (Leu16Ser and Arg313His), while G81T (Ala27Ala) is a synonymous mutation. All three mutations of mutant MT2 located in the MPH coding region (T806A, C821T, and C892T), resulting in the amino acid substitutions of Ile269Ser, Ala274Val, and Pro298Ser, respectively.

To determine the contribution of each mutation to the enhanced extracellular MPH activity in mutant MT1 and MT2, each mutation was individually introduced into strain BPC1, generating mutants of MT-16, MT-27, MT-269, MT-274, MT-298, and MT-313. All six mutations did not affect the growth of their hosts compared with strain BPC1 (data not shown). The extracellular MPH activity of mutants MT-16, MT-27, MT-269, MT-298, and MT-313 were 1.73-, 1.40-, 1.75-, 1.48-, and 2.03-fold that of strain BPC1, respectively, while mutant MT-274 exhibited a little bit lower extracellular MPH activity compared with strain BPC1 (Figure 4B). The extracellular MPH secreted by mutant MT-16, MT-27, MT-269, MT-298, and MT-313 were purified through Ni-NTA affinity chromatography individually. The MPH protein yield from mutant MT-269, MT-298, and MT-313 were close to that from strain BPC1, while the MPH protein yield from mutant MT-16 and MT-27 were 1.70- and 1.46-fold that from strain BPC1 (Figure 4D). Moreover, the specific activity of MPH variant T806A, C892T, and G938A were 1.53-, 1.43-, and 1.52-fold of wild-type MPH, respectively, while the specific activity of MPH variant T47C and G81T was comparable with that of wild-type MPH (Figure 4C). Taken together, mutation T47C and G81T in the SP_*AprE*_ coding region enhanced extracellular MPH activity through increasing MPH protein yield, while the contribution of mutation T806A, C892T, and G938A was enhancing the catalytic activity of MPH against chlorpyrifos. Additionally, to further elevate the extracellular MPH activity, two new mutants were created by recombining the mutations. Mutant MT-C2 harbors mutations T47C and T806A, and mutant MT-C3 contains mutations of T47C, T806A, and G938A. The extracellular MPH activities of mutant MT-C2 and MT-C3 were 2.78- and 3.32-fold that of strain BPC1 (Figures 4E,F), respectively, indicating that the contribution of these mutations could be accumulated when combined.

## Discussion

In the present work, to efficiently insert epPCR products into the chromosome of *B. subtilis*, we first employed a PCR based multimerization method to fuse epPCR products with the flanking region and Ab^*R*^. The insertion construct was then transformed into supercompetent cells using a modified protocol, generating 5.31 × 10^5^ transformations/μg DNA. The library was created within 1 day and is large enough for the needs of directed evolution (10^4^–10^5^ mutants are usually screened in directed evolution). Although the library generated here is smaller than those constructed by the multimeric plasmid method (Zhang et al., 2011; Li X. Z. et al., 2017), our method solves the problems of plasmid instability and heterozygosity encountered in the plasmid based method.

A crucial step in the present method is the assembly of the insertion construct (LF-Ab^*R*^-GOI-RF). We attempted to fuse the three fragments (LF-Ab^*R*^, GOI, and RF) through PCR extension and Gibson assembly, but both methods resulted in a low yield of insertion construct. By contrast, the PCR based multimerization method (Shafikhani et al., 1997; You et al., 2012) realized the efficient assembly of the insertion construct (Figure 2B). Moreover, we demonstrated that the transformation of the insertion construct could be substantially enhanced by raising the competent cell amount, expressing the recombination-promoting protein, as well as increasing flanking region size (Figures 2C–E). NgAgo-like proteins were found to be able to improve homologous recombination by interacting with RecA in bacteria (Fu et al., 2019; Wu et al., 2020). The transformation of the insertion construct may be further optimized through testing different NgAgo-like proteins in future studies.

Plasmid or chromosome is used to carry the expression cassette when producing a protein in *B. subtilis*. The plasmid mediated protein expression has the limitations of plasmid instability and safety concern (the use of Ab^*R*^) (Bron et al., 1991), while the chromosomal integration manner can overcome the limitations of plasmid manner, but the low gene dose usually leads to a low protein yield. Therefore, the transcription and translation level of GOI needs to be dramatically enhanced to achieve high protein yield in an integrated manner. Mutant MT1 harbors two mutations in the SP_*AprE*_ coding region, both of which could improve the yield of MPH (Figures 4B,D). Nijland et al. (2007) have reported that a single amino acid change can remarkably increase the secretion level of β-toxin. Therefore, the random mutagenesis strategy established here could be applied in improving the protein secretion level in *B. subtilis*.

## Data Availability Statement

The raw data supporting the conclusions of this article will be made available by the authors, without undue reservation.

## Author Contributions

XYan developed the project idea and revised the manuscript. BY performed most of the experiments, analyzed data, and prepared the manuscript. YL, QT, and XYao did some data analysis and performed some experiments. MC provided consultation for the work and contributed significantly to the preparation of the manuscript. All authors reviewed and agreed on the content of the final manuscript.

## Conflict of Interest

The authors declare that the research was conducted in the absence of any commercial or financial relationships that could be construed as a potential conflict of interest.
